# Genomic Region Containing Toll-Like Receptor Genes Has a Major Impact on Total IgM Antibodies Including KLH-Binding IgM Natural Antibodies in Chickens

**DOI:** 10.3389/fimmu.2017.01879

**Published:** 2018-01-09

**Authors:** Tom V. L. Berghof, Marleen H. P. W. Visker, Joop A. J. Arts, Henk K. Parmentier, Jan J. van der Poel, Addie L. J. Vereijken, Henk Bovenhuis

**Affiliations:** ^1^Animal Breeding and Genomics, Department of Animal Sciences, Wageningen University & Research, Wageningen, Netherlands; ^2^Adaptation Physiology, Department of Animal Sciences, Wageningen University & Research, Wageningen, Netherlands; ^3^Hendrix Genetics Research, Technology and Services B.V., Research & Technology Centre, Boxmeer, Netherlands

**Keywords:** natural antibody, antibody concentration, genetic parameter, heritability, genome-wide association study, B cell development, B cell maturation, B cell survival

## Abstract

Natural antibodies (NAb) are antigen binding antibodies present in individuals without a previous exposure to this antigen. Keyhole limpet hemocyanin (KLH)-binding NAb levels were previously associated with survival in chickens. This suggests that selective breeding for KLH-binding NAb may increase survival by means of improved general disease resistance. Genome-wide association studies (GWAS) were performed to identify genes underlying genetic variation in NAb levels. The studied population consisted of 1,628 adolescent layer chickens with observations for titers of KLH-binding NAb of the isotypes IgM, IgA, IgG, the total KLH-binding (IgT) NAb titers, total antibody concentrations of the isotypes IgM, IgA, IgG, and the total antibodies concentration in plasma. GWAS were performed using 57,636 single-nucleotide polymorphisms (SNP). One chromosomal region on chromosome 4 was associated with KLH-binding IgT NAb, and total IgM concentration, and especially with KLH-binding IgM NAb. The region of interest was fine mapped by imputing the region of the study population to whole genome sequence, and subsequently performing an association study using the imputed sequence variants. 16 candidate genes were identified, of which *FAM114A1*, Toll-like receptor 1 family member B (*TLR1B*), *TLR1A*, Krüppel-like factor 3 (*KLF3*) showed the strongest associations. SNP located in coding regions of the candidate genes were checked for predicted changes in protein functioning. One SNP (at 69,965,939 base pairs) received the maximum impact score from two independent prediction tools, which makes this SNP the most likely causal variant. This SNP is located in *TLR1A*, which suggests a fundamental role of TLR1A on regulation of IgM levels (i.e., KLH-binding IgM NAb, and total IgM concentration), or B cells biology, or both. This study contributes to increased understanding of (genetic) regulation of KLH-binding NAb levels, and total antibody concentrations.

## Introduction

Modern poultry production is facing high impact changes in production systems and management (1, 2). These changes may enhance the risks of infectious disease, because of higher pathogenic pressure. According to FAO statistics, economic losses in poultry production due to diseases were as high as 10–20% of the gross production value in developed countries in 2014.^1^ Only few years ago, diseases were controlled by (preventive and abundant) use of antibiotics, but this resulted in an increased risk for antibiotic resistance. New sustainable strategies for obtaining robust populations need to be developed, in addition to vaccination strategies, and feed formulations. Selective breeding for an improved immune system might be an important additional strategy to improve general disease resistance (3).

Selective breeding for an improved immune system requires a trait that is heritable, easy to measure, and preferentially related to general disease resistance (4). Natural antibodies (NAb) might be a good candidate trait. NAb are defined as antigen-binding antibodies present in individuals without a previous exposure to this antigen (5). NAb play an essential role in the innate, and adaptive immunity against self-antigens, and various types of pathogens, and are contributing to healthy aging, and disease resistance (6–8). Different NAb isotypes are found: predominantly IgM, but also IgA, and IgG (8, 9). In earlier studies, high NAb titers (especially IgM) binding the overt antigen keyhole limpet hemocyanin (KLH) at adolescence, but not at later age, were related to lower mortality of commercial layer chickens (10–12). KLH-binding NAb levels at adolescence were previously estimated to have heritabilities between 0.07 and 0.14 in white layer chickens (4). In addition, some genomic regions underlying genetic variation of NAb titers were identified by using dedicated single-nucleotide polymorphism (SNP) sets with 1,022 SNP (13, 14). However, to the best of our knowledge, no genome-wide association studies (GWAS) for NAb levels in chicken have been reported.

This study describes the first GWAS on NAb in chicken. The studied population consisted of 1,628 adolescent purebred White Leghorn chickens with observations for KLH-binding NAb titers of the isotypes IgM, IgA, IgG, and total KLH-binding NAb titers (IgT). In addition, phenotypes on total antibody concentrations of the isotypes IgM [total IgM (tIgM)], IgA (tIgA), IgG (tIgG), and total antibody (tIgT) concentration were collected to investigate similarities/differences between KLH-binding NAb, and total antibody concentration. Identification of associated genomic regions was performed by using 57,636 SNP in a single SNP GWAS. Regions were fine mapped by performing an association study using imputed genotypes based on sequence data with the aim to identify candidate gene(s), and possibly the causal variant(s). The role of the found association(s) in antibody-related immunity in chickens will be discussed.

## Materials and Methods

### Ethics Approval Statement

Collection of samples and data was done according to Hendrix Genetics (HG) protocols, under the supervision of HG employees. Samples and data were collected as part of routine data collection in a commercial breeding program for layer chickens in The Netherlands. Samples and data were collected on a breeding nucleus of HG for breeding purposes only and are a non-experimental, agricultural practice, regulated by the Act Animals, and the Royal Decree on Procedures. The Dutch Experiments on Animals Act does not apply to non-experimental, agricultural practices. An ethical review by the Statement Animal Experiment Committee was therefore not required. No extra discomfort was caused for sample collection for the purpose of this study.

### Study Population

Samples and data were obtained from a purebred White Leghorn chicken line (in other work referred to as “WA”), which is a layer chicken line selected mainly for egg production, but also for other production traits, e.g., traits related to egg quality. The studied chicken population comprised 1,628 chickens, of which 696 males and 932 females. The study population originated from 112 sires and 288 dams. 437 chickens in the current study were also included in the study of Berghof et al. (4). Of these 437 chickens, 222 female chickens were also included in the study of van der Klein et al. (15). The chickens were kept according to standard management of breeding nucleus farms of HG. Further details can be found in van der Klein et al. (15).

The chickens received obligatory vaccinations against Marek’s disease [1 day of age intramuscular (i.m.)], infectious bronchitis (1 day of age, 12–14 days of age, 10 weeks of age, 12 weeks of age *via* spray; 16 weeks of age i.m.), Newcastle disease (13 day of age, 42 days of age, 12 weeks of age *via* spray; 16 weeks of age i.m.), infectious bursal disease (25 days of age *via* spray; 16 weeks of age i.m.), chicken anemia virus (16 weeks of age *via* water), fowl pox (16 weeks of age by wing web injection), and avian encephalomyelitis (16 weeks of age by wing web injection).

Blood of the study population was collected once between 15 and 22 weeks of age (i.e., adolescence), without anesthesia/analgesia. Samples of the study population were collected in four batches with approximately 1.75 years between the first and last batch. No chickens were killed for sample collection. The blood samples were centrifuged, and plasmas and blood cells were collected separately, and stored at −20°C until use.

### KLH-Binding NAb Titers

Optical density (OD) of KLH-binding immunoglobulins of the isotypes IgM, IgA, and IgG, and the total KLH-binding (IgT) immunoglobulins were determined in individual plasma samples by an indirect two-step ELISA as described by van der Klein et al. (15), and Berghof et al. (4). Briefly, plasma samples were 1:10 prediluted (for IgM, IgG, and IgT analyses) or were 1:5 prediluted (for IgA analysis) with dilution buffer [PBS (10.26 g/L Na_2_HPO_4_⋅H_2_O, 2.36 g/L KH_2_PO_4_, and 4.50 g/L NaCl; pH 7.2) containing 0.5% normal horse serum, and 0.05% Tween^®^ 20]. Predilutions were stored at 4°C until use the next day, or were stored at −20°C until use (with a maximum storage time of 3 months). Flat-bottomed, 96-well medium binding plates (Greiner Bio-One, Alphen a/d Rijn, The Netherlands) were coated with 2 μg/mL KLH in 100 μL coating buffer (5.3 g/L Na_2_CO_3_, and 4.2 g/L NaHCO_3_; pH 9.6) per well, and incubated at 4°C overnight. After washing for 6 s with tap water containing Tween^®^ 20, plates were tapped dry. The 1:10 predilution of the samples were further diluted in the KLH-coated plates with dilution buffer to 1:40, 1:160, 1:640, and 1:2,560 test dilutions for IgM, IgG, and IgT, 1:10, 1:20, 1:40, and 1:80 for IgA. Duplicate standard positive plasma samples (a pool of male plasmas) were stepwise 1:1 diluted with dilution buffer, and pipetted into the KLH-coated plates. A minimum of five samples per plate was maintained to ensure proper adjustment of the titers for plate effects in the statistical analyses. The plates were incubated for 1.5 h at room temperature (20–25°C). After washing, plates were incubated with 1:20,000-diluted goat-antichicken IgM labeled with peroxidase (PO) (Cat# A30-102P, RRID:AB_66857),^2^ 1:7,500-diluted goat-antichicken IgA labeled with PO (Cat# A30-103P, RRID:AB_66833) (see text footnote 2), 1:40,000-diluted goat-antichicken IgG(Fc) labeled with PO (Cat# A30-104P, RRID:AB_66843) (see text footnote 2), or 1:20,000-diluted rabbit-antichicken IgG heavy and light chain (IgT) labeled with horse radish PO (Cat# A30-107P, RRID:AB_67386) (see text footnote 2), (all polyclonal antibodies from Bethyl Laboratories, Montgomery, TX, USA), and incubated for 1.5 h at room temperature. After washing, binding of the antibodies to KLH was visualized by adding 100 μL substrate buffer [containing reverse osmosis purified water, 10% tetramethylbenzidine buffer (15.0 g/L sodium acetate and 1.43 g/L urea hydrogen peroxide; pH 5.5), and 1% tetramethylbenzidine (8 g/L TMB in DMSO)] at room temperature. After approximately 15 min, the reaction was stopped with 50 μL of 1.25 M H_2_SO_4_. OD was measured with a Multiskan Go (Thermo scientific, Breda, The Netherlands) at 450 nm.

Antibody titers were calculated as described by Frankena (16) [taken from De Koning et al. (17)]. Briefly, OD of the duplicate standard positive plasma samples were averaged for each plate. Logit values of OD per plate were calculated using:

$$\text{logit OD}=\text{ln}\left(\frac{\text{OD}}{\left({\text{OD}}_{\text{max}}-\text{OD}\right)}\right),$$

where OD is the OD of a well, and OD_max_ is the maximum averaged OD of the duplicate standard positive plasma samples. The last positive well (lpw) of the averaged duplicate standard positive plasma sample was set to the sixth dilution. A linear regression line of the logit OD against the respective log_2_-dilution values of the averaged duplicate standard positive plasma samples was determined, which resulted in regression coefficient β. Titers of plasma samples per plate were calculated using:

$$\text{titer}\;=\;\frac{{\text{logit OD}}_{\text{lpw}}\;-\;\left(\;{\text{logit OD}}_{\text{sample}}\;-\;\beta \;\times \;{\text{log}}_{\text{2}}\left({\text{dilution}}_{\text{sample}}\right)\;\right)}{\beta },$$

were logit OD_lpw_ is the estimated logit OD at the lpw calculated with the estimated linear regression function using the log_2_-dilution value of that well, logit OD_sample_ is the logit OD calculated of the OD closest to 50% of OD_max_ for a plasma sample of an individual (OD_sample_), β is the regression coefficient of the estimated linear regression function of the averaged duplicate standard positive plasma samples, and log_2_ (dilution_sample_) is the log_2_-dilution value at which OD_sample_ occurred, as described by De Koning et al. (17).

The total number of observations was 1,627 for IgM NAb, 1,608 for IgA NAb, 1,623 for IgG NAb, and 1,625 for IgT NAb (see Table 1).

**Table 1:** Descriptive statistics of KLH-binding natural antibody (NAb) titers, and total antibody concentrations (μg/ml) in an adolescent White Leghorn chicken population for KLH-binding IgM NAb (IgM) titer, KLH-binding IgA NAb (IgA) titer, KLH-binding IgG NAb (IgG) titer, total KLH-binding NAb (IgT) titer, tIgM concentration, total IgA (tIgA) concentration, total IgG (tIgG) concentration, and total antibody (tIgT) concentration.

	KLH-binding natural antibody titers				Total antibody concentrations (μg/ml)			
	IgM	IgA	IgG	IgT	tIgM	tIgA	tIgG	tIgT^a^
*n*	1,627	1,608	1,623	1,625	1,619	1,586	1,615	1,573
Mean (SD)	5.8 (1.1)	5.4 (1.2)	5.5 (1.3)	5.6 (1.3)	355 (201)	314 (143)	7,856 (3,679)	8,484 (3,741)
Range^b^	4.0–7.6	3.5–7.4	3.5–7.8	3.6–7.7	142–701	157–585	3,530–14,169	4,071–15,003
*h*^2^ (SE)	0.14^c^	0.10^c^	0.07^c^	0.12^c^	0.23 (0.05)^d^	0.22 (0.05)^d^	0.06 (0.03)^d^	0.08 (0.04)^d^
*m*^2^	0.06^c^	NS^c^	NS^c^	NS^c^	NS^d^	NS^d^	NS^d^	NS^d^

*The number of chickens with an observation per trait, the mean (and SD) of the observations, the range of the observations (5th percentile to 95th percentile), the heritability (*h*^2^) (and SE), and the maternal environmental effect (*m*^2^)*.

*^a^tIgT = tIgM + tIgA + tIgG*.

*^b^Range shows 5th percentile and 95th percentile, respectively*.

*^c^The reported NAb *h*^2^ and *m*^2^ are of a previous study (4)*.

*^d^The *h*^2^ and *m*^2^ are estimated based on a log_10_-tranformation of the data*.

### Total Antibody Concentrations

Total concentrations (μg/ml) of the immunoglobulin isotypes IgM (tIgM), IgA (tIgA), and IgG (tIgG) were determined in individual plasma samples by an indirect sandwich ELISA according to manufacturer’s protocols (IgM: Cat# E30-102; IgA: Cat# E30-103; IgG: Cat# E30-104; all from Bethyl Laboratories) with minor additions: (1) plasma samples were 1:5 prediluted, and step-wise diluted to 1:500 (for tIgA), 1:5,000 (for tIgM), and 1:50,000 (for tIgG) with sample/conjugate diluent. (2) Flat-bottomed, 96-well medium binding plates (Greiner Bio-One) were used for ELISA. (3) Washing procedure consisted of washing each well two times with 200 μl and subsequently three times with 100 μl wash solution. Plates were emptied, and tapped dry in between, and at the end. (4) 100 μl ELISA blocking solution was added to each well. (5) A minimum of five samples per plate was maintained to ensure proper adjustment of antibody concentrations for plate effects in the statistical analyses. (6) A duplicate of a standard positive plasma sample (obtained from Bethyl Laboratories) was used per plate. (7) Enzyme substrate (TMB), and ELISA stop solution were made and applied as specified for “KLH-binding natural antibody titers” (see above). (8) OD were measured with a Multiskan Go. (9) All samples on a plate were corrected for the average background OD of the two blanks. (10) SoftMax^®^ Pro 7.0 build 226962 (Free Trial) was used for generating a 4-parameter logistic curve fit based on the averages of the two standard ranges, and calculating isotype concentrations per sample.

The total concentration (μg/ml) of antibodies (tIgT) was calculated by summing the concentrations for tIgM, tIgA, and tIgG. tIgM, tIgA, tIgG, and tIgT were log_10_-tranformed to normalize the data for statistical analyses. The total number of observations was 1,619 for tIgM, 1,586 for tIgA, 1,615 for tIgG, and 1,573 for tIgT (see Table 1).

### Genotypes

DNA was extracted from the collected blood cells. The study population was genotyped for a 2,740 SNP set (*n* = 488) (Illumina, San Diego, CA, USA), or for a 52,232 SNP set from which 11,173 SNP were used (*n* = 1,140) (Illumina). Both sets were imputed with Beagle 4.0 (18) (accuracy ≥ 97%) to a 57,636 SNP set, based on approximately 120 key ancestors of this chicken line. The SNP distribution over the chicken chromosomes (based on Gallus_gallus-5.0) can be found in Table S1 in Supplementary Material.

Quality control was applied in four steps: (1) All monomorphic SNP were removed. (2) SNP with at least one genotype class with less than nine chickens with phenotypic observations (~0.5% of the population) were removed. (3) SNP on autosomes with a strong deviation from Hardy Weinberg Equilibrium ${\text{(χ}}_{1}^{2}\text{-value}\geq \text{600)}$ were removed. (4) SNP on the allosome Z with a strong deviation from Hardy Weinberg Equilibrium ${\text{(χ}}_{1}^{2}\text{-value}\geq \text{600)}$ in males were removed for the whole study population. These criteria were chosen to exclude SNP which are likely to have a high rate of genotyping error and to exclude very low frequency SNP from the data set (19).

### Genetic Parameters

The additive genetic variances ($\sigma_{\text{a}}^{2}$), and the maternal environmental variance ($\sigma_{\text{m}}^{2}$) for KLH-binding NAb titers were previously estimated in a similar, but larger population of this chicken line (4) (see Table 1). The additive genetic variances ($\sigma_{\text{a}}^{2}$), and heritabilities for tIgM, tIgA, tIgG, and tIgT concentrations were estimated with model 1, in a similar approach as for the NAb titers described by Berghof et al. (4).

Model 1 was

(1) $${\text{y}}_{\mathrm{ij}}=\mu +{\text{P}}_{i}+\beta_{\text{1}}\times {\text{Age}}_{\mathrm{ij}}+{\text{id}}_{j}+{\text{e}}_{\mathrm{ij}},$$

where y*_ij_* is the tIgM concentration, tIgA concentration, tIgG concentration, or tIgT concentration, μ is the overall mean, P*_i_* is the fixed effect of plate on which a sample was analyzed (*i* = 1–26 for tIgM and tIgA, or *i* = 1–27 for tIgG and tIgT), Age*_ij_* is the covariate describing the effect of age at sampling (15–22 weeks of age) with regression coefficient β_1_, id*_j_* is the random additive genetic effect of the *j*th chicken assumed to be distributed as $\sim \text{N}\left(0,\text{A}\sigma_{\text{a}}^{2}\right)$, and e*_ij_* is the residual term assumed to be distributed as $\sim \text{N}\left(0,\text{I}\sigma_{\text{e}}^{2}\right)$. Assumed (co)variance structures of the random model terms are ${\text{A}\sigma }_{\text{a}}^{2}$ and ${\text{I}\sigma }_{\text{e}}^{2}$, in which **A** is the additive genetic relationship matrix, $\sigma_{\text{a}}^{2}$ is the additive genetic variance, **I** is an identity matrix, and $\sigma_{\text{e}}^{2}$ is the residual variance. The pedigree used to construct **A** consisted of 2,537 individuals, and was based on a minimum of three generations of ancestors for each individual in the study population. The estimated plate effect also corrects for (confounded) effects on the samples, such as sex and storage. The estimated regression coefficient of age also corrects (partly) for (confounded) effects of vaccinations at 16 weeks of age.

Model 1 extended with a random maternal environmental effect [for IgM NAb only, see Berghof et al. (4)], were also used to estimate genetic, and phenotypic correlations with bivariate analyses, as described by Berghof et al. (4). The genetic correlations give estimates on the extent to which two traits are influenced by the same genetic variations (i.e., genes). The phenotypic correlations give estimates on the extent to which two traits are influenced by the same genetic variation, and the same environmental variation (e.g., position of cage in the stable, temperature, age). See also Stear et al. (20) for a detailed explanation.

The statistical analyses were performed using ASReml^®^ 4.1 (21).

### Genome-Wide Association Studies

A single-SNP linear animal model was used for studying KLH-binding IgM, IgA, IgG, and IgT NAb titers and tIgM, tIgA, tIgG, and tIgT concentrations associations.

All traits, except IgM NAb, were analyzed using model 2a:

(2a) $${\text{y}}_{\mathrm{ijk}}=\mu +{\text{P}}_{i}+\beta_{\text{1}}\times {\text{Age}}_{\mathrm{ijk}}+{\text{SNP}}_{j}+{\text{id}}_{k}+{\text{e}}_{\mathrm{ijk}},$$

and IgM NAb was analyzed using model 2b:

(2b) $${\text{y}}_{\mathrm{ijkl}}=\mu +{\text{P}}_{i}+\beta_{\text{1}}\times {\text{Age}}_{\mathrm{ijkl}}+{\text{SNP}}_{j}+{\text{id}}_{k}+{\text{d}}_{l}+{\text{e}}_{\mathrm{ijkl}},$$

where y_*ijk*_ is the IgA NAb titer, IgG NAb titer, IgT NAb titer, tIgM concentration, tIgA concentration, tIgG concentration, or tIgT concentration, y*_ijkl_* is the IgM NAb titer, μ is the overall mean, P*_i_* is the fixed effect of plate on which a sample was analyzed (*i* = 1–91 for IgM NAb, IgG NAb, and IgT NAb; *i* = 1–100 for IgA NAb; *i* = 1–26 for tIgM; and tIgA, or *i* = 1–27 for tIgG, and tIgT), Age*_ijk_* and Age*_ijkl_* are the covariates describing the effect of age at sampling (15–22 weeks of age) with regression coefficient β_1_, SNP*_j_* is the fixed effect of genotype class *j* (*j* = AA, AB, or BB) of the analyzed SNP, id*_k_* is the random additive genetic effect of the *k*th chicken assumed to be distributed as $\sim \text{N}\left(0,\text{A}\sigma_{\text{a}}^{2}\right)$, d*_l_* is the random effect of the *l*th dam (IgM NAb only) assumed to be distributed as $\sim \text{N}\left(0,\text{I}\sigma_{\text{m}}^{2}\right)$, and e*_ijk_* and e*_ijkl_* are the residual terms assumed to be distributed as $\sim \text{N}\left(0,\text{I}\sigma_{\text{e}}^{2}\right)$. Assumed (co)variance structures of the random model terms are ${\text{A}\sigma }_{\text{a}}^{2}$, and ${\text{I}\sigma }_{\text{e}}^{2}$ (for all), and ${\text{I}\sigma }_{\text{m}}^{2}$ (for IgM NAb only), in which **A** is the additive genetic relationship matrix, $\sigma_{\text{a}}^{2}$ is the additive genetic variance, **I** are identity matrices, $\sigma_{\text{e}}^{2}$ is the residual variance, and $\sigma_{\text{m}}^{2}$ is the maternal environmental variance (for IgM NAb only).

The variances for random factors were fixed to values obtained from analyses from models without a SNP effect: the additive genetic variances ($\sigma_{\text{a}}^{2}$) and the maternal environmental variance ($\sigma_{\text{m}}^{2}$) for NAb titers were fixed to values estimated by Berghof et al. (4) in a similar population of this chicken line. These estimates are more accurate, because the studied population was bigger. The additive genetic variances ($\sigma_{\text{a}}^{2}$) for tIgM, tIgA, tIgG, and tIgT, concentrations were estimated with model 1.

The pedigree used to construct **A** consisted of 2,537 individuals, and was based on a minimum of three generations of ancestors for each individual in the study population. The estimated plate effect also corrects for (confounded) effects on the samples, such as sex and storage.

The statistical analyses were performed using ASReml^®^ 4.1 (21).

*p*-values of SNP effects were checked for inflation (22), which was expressed as inflation factor λ (23). λ was estimated using the “estlambda()” function of the R package “GenABEL” (24). Genomic control was applied when λ ≥ 1.1 [Wellcome Trust Case Control Consortium (25), taken from Duijvesteijn et al. (26)] by dividing the test statistics (*F*-values) by λ, and subsequently recalculating the *p*-values. A genome-wide false discovery rate (FDR) was estimated based on observed *p*-values using the R package “qvalue” (27). A FDR ≤ 0.05 was used to indicate significant associations, and a FDR ≤ 0.20 was used to indicate suggestive associations. The lead SNP [SNP with highest −log_10_(*p*-value)] variance as a percentage of the additive genetic variance within an associated region was estimated as $\sigma_{\text{SNP}}^{2}∕\sigma_{\text{a}}^{2}\times 100\%$, where $\sigma_{\text{SNP}}^{2}$ was calculated based on estimated SNP effects from model 2a for all, except IgM NAb, or model 2b for IgM NAb and the genotypic frequencies. Significant differences between genotype classes were tested with a *T*-test using the “!TDIFF” statement in ASReml^®^ 4.1 (21). A *p*-value ≤0.05 was considered to be significant, and a *p*-value ≤0.10 was considered to be suggestive.

### Fine Mapping, and Identification of Possible Causal Variant

The chickens were imputated to full genome sequence in two steps based on the full genome sequences of 70 key ancestors of this chicken line. Imputation was done with Beagle 4.0 (18) (accuracy ≥ 88%) by imputing the 57,636 SNP set (for the 2,740 SNP set), or the 52,232 SNP set to full genome sequence with an average sequence depth of 12.4 (and SD of 2.1).

Associated genomic regions were selected for fine mapping, if at least 1 SNP was significant (FDR ≤ 0.05). Fine mapping regions were defined based on the last significant SNP on the border of significantly associated regions. The fine mapping regions consisted of the first SNP outside the significantly associated regions −5 and +5 Mbp.

Quality control of the imputed SNP that were used in the fine mapping studies, was applied in four steps as described above. Statistical analyses for the selected regions were performed as described above. Genomic control was applied using λ values from previous analyses, to be able to compare between analyses.

Single-nucleotide polymorphism of candidate genes in the fine mapped regions were checked for amino acid substitutions and predicted changes in protein functioning as a result of the nucleotide change in coding regions by using the Ensembl Variant Effect Predictor (28), including the integrated SIFT option (29), and by using PolyPhen-2 (30).

## Results

In total 1,628 chickens, of which 696 males, and 932 females, were in the study population. Descriptive statistics and genetic parameters are shown in Table 1. Mean titers for KLH-binding NAb were 5.8 for IgM, 5.4 for IgA, 5.5 for IgG, and 5.6 for total KLH-binding NAb (IgT). Mean concentrations for total antibody concentrations were 355 μg/ml for tIgM, 314 μg/ml for tIgA, 7,856 μg/ml for tIgG, and 8,484 μg/ml for total antibody concentration (tIgT). Heritabilities for total antibody concentrations were estimated to be 0.23 for tIgM, 0.22 for tIgA, 0.06 for tIgG, and 0.08 for tIgT. Maternal environmental effects were not significant for any of the total antibody concentrations. Age was not significant for tIgA, and tIgT but was kept in the model to be consistent with the other analyses. Heritabilities and maternal environmental variances ($\sigma_{\text{m}}^{2}$) for NAb were previously estimated in a similar, but larger population of this chicken line (see Table 1) (4).

Genetic, and phenotypic correlations between the corresponding types of KLH-binding NAb titers and total antibody concentrations are shown in Table 2. Genetic correlations were: 0.91 for IgM NAb/tIgM, 0.38 for IgA NAb/tIgA, −0.61 for IgG NAb/tIgG, and −0.27 for IgT NAb/tIgT. Phenotypic correlations were: 0.41 for IgM NAb/tIgM, 0.26 for IgA NAb/tIgA, 0.08 for IgG NAb/tIgG, and 0.03 for IgT NAb/tIgT. In addition, genetic and phenotypic correlations between all total antibody concentrations were estimated (see Table S2 in Supplementary Material).

**Table 2:** Estimated genetic correlations, and phenotypic correlations of KLH-binding natural antibody (NAb) titers and total antibody concentrations (μg/ml) in an adolescent White Leghorn chicken population.

	Genetic correlation	Phenotypic correlation
IgM^a^/tIgM	0.91 (0.06)	0.41 (0.02)
IgA/tIgA	0.38 (0.22)	0.26 (0.03)
IgG/tIgG	−0.61 (0.55)	0.08 (0.03)
IgT/tIgT^b^	−0.27 (0.31)	0.03 (0.03)

*The correlations between: KLH-binding IgM NAb (IgM) titer and total IgM (tIgM) concentration; KLH-binding IgA NAb (IgA) titer and total IgA (tIgA) concentration; KLH-binding IgG NAb (IgG) titer and total IgG (tIgG) concentration; and total KLH-binding NAb (IgT) titer and total antibody (tIgT) concentration. tIgM, tIgA, tIgG, and tIgT were log_10_-tranformed for the analyses. SE is shown in parentheses*.

*^a^Dam component was fixed at boundary (0) in ASReml analysis*.

*^b^tIgT = tIgM + tIgA + tIgG*.

**Figure 1:**
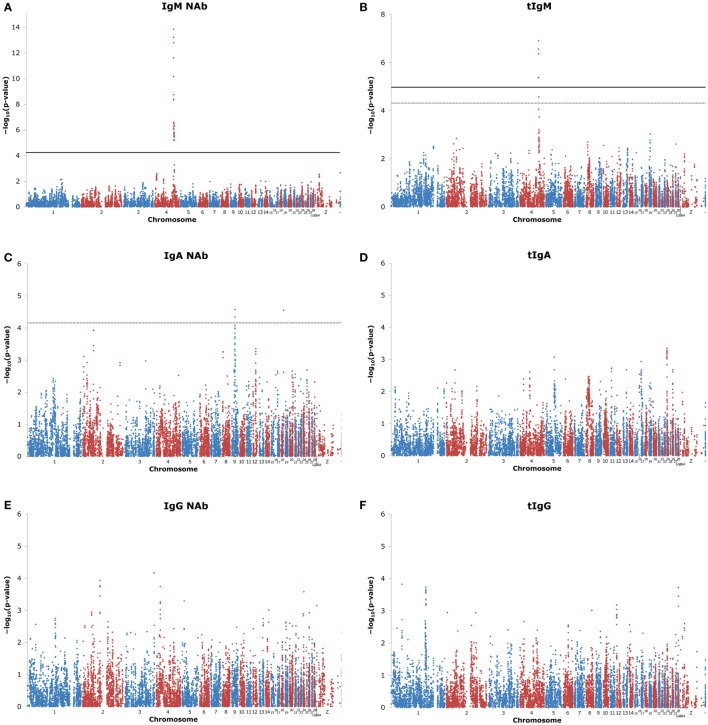

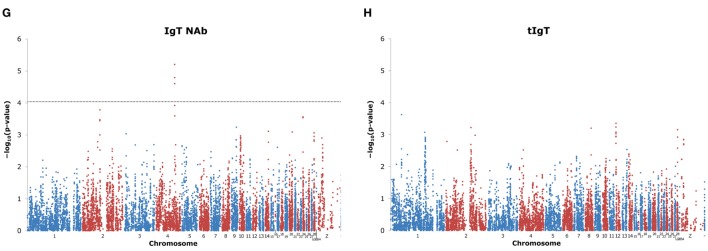
Manhattan plots of genome-wide association studies (GWAS) for keyhole limpet hemocyanin (KLH)-binding natural antibody (NAb) titers, and total antibody concentrations (μg/ml) in an adolescent White Leghorn chicken population. The figures show GWAS results for: **(A)** KLH-binding IgM NAb (IgM) titer; **(B)** total IgM (tIgM) concentration; **(C)** KLH-binding IgA NAb (IgA) titer; **(D)** total IgA (tIgA) concentration; **(E)** KLH-binding IgG NAb (IgG) titer; **(F)** total IgG (tIgG) concentration; **(G)** total KLH-binding NAb (IgT) titer; and **(H)** total antibody (tIgT) concentration. tIgM, tIgA, tIgG, and tIgT were log_10_-tranformed for the analyses. Genomic control was applied to test statistics of IgM NAb, tIgM, tIgA, tIgG, and tIgT. On the *x*-axis is the physical position of the 15,579 used SNP across the chicken genome (chromosomes 1–28, linkage groups LGE64, chromosome Z, and unplaced represented by –). Positions are based on Gallus_gallus-5.0. On the *y*-axis is the −log_10_(*p*-value_SNP_) for association of the SNP with the investigated trait. Each dot represents one SNP. Note the different *y*-axes for IgM and tIgM. The false discovery rate (FDR) threshold was set at 0.05 for significant SNP (solid horizontal line) and at 0.20 for suggestive SNP (dashed horizontal line). If no SNP reached the FDR threshold, the threshold could not be estimated. For IgM, no −log_10_(*p*-value_SNP_) reached 0.05 ≤ FDR ≤ 0.20, and therefore, FDR ≤ 0.20 is not shown.

Quality control of the SNP resulted in removal of 37,053 SNP, because they were monomorphic (step 1), and an additional removal of 5,005 SNP, because of a low number of observations per genotype class or a strong deviation from Hardy-Weinberg equilibrium (steps 2–4) (see Table S1 in Supplementary Material). In total, 15,579 SNP were used for GWAS, though the actual number of used SNP per trait varied between 15,431 and 15,578 SNP due to missing phenotypes. Figure 1 shows the “Manhattan plots” of the GWAS. Results shown for KLH-binding IgM NAb, and all total antibody concentrations (tIgM, tIgA, tIgG, and tIgT) were adjusted for inflation factor λ, which was estimated to be 1.67 for IgM NAb, 1.23 for tIgM, 1.27 for tIgA, 1.13 for tIgG, and 1.14 for tIgT. This indicates a deviation of the *p*-values from their expected distribution under the null hypotheses of no genetic association. After correcting the test statistics, and subsequently recalculating the *p*-values, all new λ were below 1. Inflation factors for IgA NAb, IgG NAb, and IgT NAb were below 1.1, and required no adjustment. For KLH-binding IgA NAb, and KLH-binding IgT NAb, none of the SNP were significantly associated (FDR ≤ 0.05), but some regions showed suggestive associations (FDR ≤ 0.20) (see Table S3 in Supplementary Material). For KLH-binding IgM NAb, and tIgM several SNP were significantly associated (see Table S3 in Supplementary Material). For KLH-binding IgG NAb, tIgA, tIgG, and tIgT no significant or suggestive associations were detected.

Tables 3 and 4 characterize the significantly, and suggestively associated genomic regions based on the GWAS results. The associated regions, and additional information can be found in Table 3. The lead SNP and additional information can be found in Table 4. Table S3 in Supplementary Material gives an overview of associated genotyped and imputed SNP.

**Table 3:** Genomic regions significantly, or suggestively associated with KLH-binding natural antibody (NAb) titers, and total antibody concentrations (μg/ml) in an adolescent White Leghorn chicken population.

Trait	Chromosome	Start (bp)–Stop (bp)	# SNP	
			Significant	Suggestive
IgM	4	69,587,709–73,362,701	35	0
IgT		69,587,709–69,814,286	0	3
tIgM		69,587,709–70,109,615	4	1
IgA	9	12,230,193–12,261,658	0	2
IgA	18	9,984,590–10,154,530	0	3

*Analyzed traits were KLH-binding IgM NAb (IgM) titer, KLH-binding IgA NAb (IgA) titer, KLH-binding IgG NAb (IgG) titer, total KLH-binding NAb (IgT) titer, total IgM (tIgM) concentration, total IgA (tIgA) concentration, total IgG (tIgG) concentration, and total antibody (tIgT) concentration. tIgM, tIgA, tIgG, and tIgT were log_10_-tranformed for the analyses. The table shows: trait, chromosome of associated genomic region, base pair (bp) start–bp stop, number of significant [false discovery rate (FDR) ≤ 0.05] SNP associated, and number of suggestive (0.05 ≤ FDR ≤ 0.20) SNP associated. Positions are based on Gallus_gallus-5.0*.

**Table 4:** Lead SNP (SNP with highest *p*-value) within genomic regions significantly, or suggestively associated with KLH-binding natural antibody (NAb) titers, and total antibody concentrations (μg/ml) in an adolescent White Leghorn chicken population.

Trait	Chromosome	Position	SNP name	−log_10_(*p*)	*n_AA_*	*n_AB_*	*n_BB_*	AA (SE)^a^	AB^a^	BB (SE)^a^	$\sigma_{\text{SNP}}^{\text{2}}∕\sigma_{\text{a}}^{\text{2}}$ (%)
IgM	4	69,702,481	rs15614874	13.84^b^	477	824	326	−0.58^y^ (0.06)	0^z^	0.01^z^ (0.06)	57.6
IgT		69,587,709	rs313004783	*5.21*	480	807	338	−0.34^y^ (0.08)	0^z^	0.09^yz^ (0.08)	16.0
tIgM		69,814,286	rs313437715	6.90^b^	465	821	333	−40.7^y,^^c^	0^z^	6.2^z,^^c^	14.1
IgA	9	12,261,658	rs15969591	*4.58*	814	708	86	0.18 (0.06)	0	0.53 (0.13)	13.5
IgA^d^	18	10,131,465	rs10731438	*4.55*	1,133	430	45	−0.05^y^ (0.07)	0^y^	0.78^z^ (0.18)	0.2

*Analyzed traits were KLH-binding IgM NAb (IgM) titer, KLH-binding IgA NAb (IgA) titer, KLH-binding IgG NAb (IgG) titer, total KLH-binding NAb (IgT) titer, total IgM (tIgM) concentration, total IgA (tIgA) concentration, total IgG (tIgG) concentration, and total antibody (tIgT) concentration. tIgM, tIgA, tIgG, and tIgT were log_10_-tranformed for the analyses. The table shows: trait, chromosome of associated genomic region, position of lead SNP, name of lead SNP, −log_10_(*p*-value_lead SNP_) (italic for suggestive association), number of chickens per genotype class, effect of genotype classes (and SE) in titer points for NAb, or μg/ml for total antibody concentrations, and percentage of additive genetic variance explained by the lead SNP ($\sigma_{\text{SNP}}^{\text{2}}∕\sigma_{\text{a}}^{\text{2}}\times 100\%$). Genotype classes with significantly (*p*-value ≤ 0.05) different effects are indicated by different superscripts (y or z). Positions are based on Gallus_gallus-5.0*.

*^a^Genotype effects are compared to the AB genotype class, which was set to 0 (zero)*.

*^b^Genomic control was applied to the test statistic*.

*^c^The estimated effects are reported in μg/ml but were calculated from the predicted values on a log_10_-scale. Predicted values were 2.43 for AA, 2.49 for AB, and 2.50 for BB. Estimated effects based on log_10_-analysis were −0.06 (0.01) for AA and 0.01 (0.01) for BB*.

*^d^Region contains three SNP with similar −log_10_(*p*-value_SNP_). The genotyped SNP is reported*.

One genomic region showed an association with KLH-binding IgT NAb, and tIgM and showed the strongest association for KLH-binding IgM NAb. The genomic region was located on chromosome 4 around 70 M base pairs (bp), and consisted of 3 suggestive SNP for IgT NAb, 4 significant, and 1 suggestive SNP for tIgM, and 35 significant SNP for IgM NAb. The genomic region on chromosome 4 had its lead SNP for IgT NAb on 69,587,709 bp (rs313004783), for tIgM on 69,814,286 bp (rs313437715), for IgM NAb on 69,702,481 bp (rs15614874). Remarkably, the heterozygous genotype class was not significantly different from one of the homozygous genotype classes, indicating full dominance. The lead SNP variance as a percentage of the additive genetic variance was approximately 16.0% for KLH-binding IgT NAb, 14.1% for tIgM, and 57.6% for KLH-binding IgM NAb, illustrating that the genomic region on chromosome 4 has a major effect on the genetic variation of KLH-binding IgT NAb, tIgM, and especially on KLH-binding IgM NAb.

Two genomic regions were detected for KLH-binding IgA NAb. The first region was located on chromosome 9 around 12 Mbp and consisted of two suggestive SNP. The lead SNP was located at 12,261,658 bp (rs15969591). The lead SNP variance was approximately 13.5% of the additive genetic variance for IgA NAb. The second region was located on chromosome 18 around 10 Mbp. This region consisted of three suggestive neighboring SNP with equal *p*-values. The region showed complete dominance. The variance of the region was approximately 0.2% of the additive genetic variance for IgA NAb.

**Figure 2:**
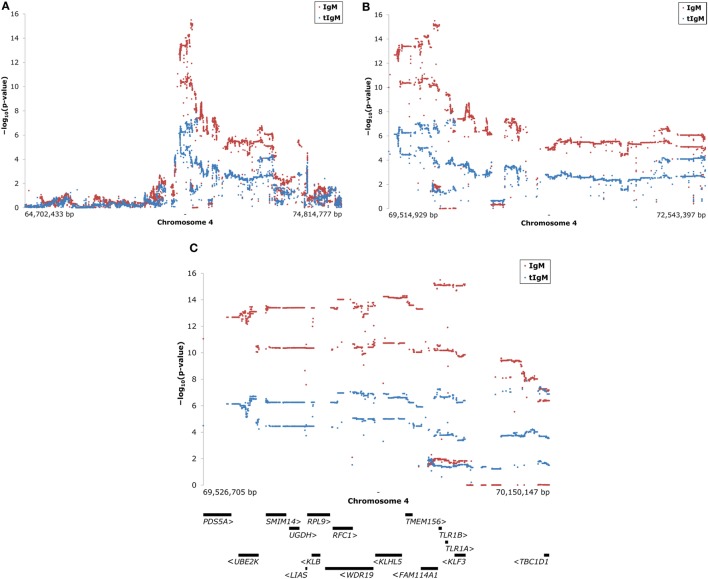
Manhattan plots of association studies of the selected region on chromosome 4 for keyhole limpet hemocyanin (KLH)-binding IgM NAb (IgM) titer in red and total IgM (tIgM) concentration (log_10_-tranformed for the analyses) in blue in an adolescent White Leghorn chicken population. On the *y*-axes is the −log_10_(*p*-value_SNP_) for the association of the investigated SNP with IgM, and tIgM after genomic control was applied. Each dot represents one SNP. The figures show: **(A)** on the *x*-axis is the physical position of the approximately 40,000 SNP used on chromosome 4 for the selected region; **(B)** zoom of peak-region in figure A [−log_10_(*p*-value_SNP_) ≥ 5]; **(C)** zoom of peak-region in figure B [−log_10_(*p*-value_SNP_) ≥ 10 for IgM and −log_10_(*p*-value_SNP_) ≥ 6 for tIgM], including candidate gene overview (32). Positions are based on Gallus_gallus-5.0. Arrows (“ <” and “ >”) indicate direction of the genes. Abbreviations genes: *PDS5A*, PDS5 cohesin-associated factor A; *UBE2K*, ubiquitin conjugating enzyme E2 K; *SMIM14*, small integral membrane protein 14; *UGDH*, UDP-glucose 6-dehydrogenase; *LIAS*, lipoic acid synthetase; *RPL9*, ribosomal protein L9; *KLB*, klotho beta; *WDR19*, WD repeat domain 19; *RFC1*, replication factor C subunit 1; *KLHL5*, kelch like family member 5; *TMEM156*, transmembrane protein 156; *FAM114A1*, family with sequence similarity 114 member A1; *TLR1B*, Toll-like receptor 1 family member B; *TLR1A*, Toll-like receptor 1 family member A; *KLF3*, Kruppel-like factor 3; *TBC1D1*, TBC1 domain family member 1 [from NCBI (31) and Ensembl (Release 87) (32)].

The significantly associated region on chromosome 4 was imputed to whole genome sequence. The region selected for further investigation ranged from approximately 64.7to 74.8 Mbp. After quality control 43,675 SNP remained for KLH-binding IgM NAb, and 39,792 SNP for tIgM, deviating because of missing phenotypes for tIgM. Figure 2 shows the results of the association study for IgM NAb, and tIgM of the selected region on chromosome 4. The highest signal for IgM NAb, and tIgM was detected in the region 69.5–70.2 Mbp with 3,153 SNP (see Figure 2C). Candidate genes (5′–3′ direction) in this region are: PDS5 cohesin-associated factor A (*PDS5A*), ubiquitin conjugating enzyme E2 K (*UBE2K*), small integral membrane protein 14 (*SMIM14*), UDP-glucose 6-dehydrogenase (*UGDH*), lipoic acid synthetase (*LIAS*), ribosomal protein L9 (*RPL9*), klotho beta (*KLB*), WD repeat domain 19 (*WDR19*), replication factor C subunit 1 (*RFC1*), kelch like family member 5 (*KLHL5*), transmembrane protein 156 (*TMEM156*), family with sequence similarity 114 member A1 (*FAM114A1*), Toll-like receptor 1 family member B (*TLR1B*), Toll-like receptor 1 family member A (*TLR1A*), Kruppel like factor 3 (*KLF3*), and TBC1 domain family member 1 (*TBC1D1*) [from NCBI (31), and Ensembl (Release 87) (32)] (see Figure 2C). The strongest associated subregion contained *FAM114A1*, *TLR1B*, *TLR1A*, and *KLF3*.

The 3,153 SNP in this region were checked for amino acid substitutions in coding regions, and consequential changes in protein functioning as a result of the nucleotide change. The Ensembl Variant Effect Predictor predicted 28 SNP to result in different amino acids. All 28 SNP could be predicted with SIFT, PolyPhen-2 or both. SIFT predicted amino acid substitutions to be “tolerated” for 11 SNP, and “deleterious” for 2 SNP. The remaining 15 SNP could not be predicted. PolyPhen-2 predicted amino acid substitution to be “benign” for 22 SNP, “possibly damaging” for 1 SNP, and “probably damaging” for 1 SNP. SIFT and PolyPhen-2 gave both the maximum impact score (“deleterious,” and “probably damaging”) to 1 SNP (69,965,939 bp), which is considered the most likely candidate as causal variant for the major effects on KLH-binding IgT NAb, tIgM, and especially on KLH-binding IgM NAb. This SNP consists of a cytosine (C)/guanine (G) polymorphism, and results in a phenylalanine (F)/leucine (L) amino acid substitution in *TLR1A* at protein position 126. The C-variant had an allele frequency of 0.45, and the G-variant had an allele frequency of 0.55. Based on Figure 1 from Keestra et al. (33), the SNP is located in leucine-rich repeat (LRR) 4.

Significance and effects of the predicted causal variant on all measured traits can be found in Table 5. KLH-binding IgM NAb, KLH-binding IgG NAb, KLH-binding IgT NAb, and tIgM showed a significant effect of the predicted causal variant. For these traits (except IgG NAb), the GG genotype class had significantly lower phenotypic values than the CG and CC genotype classes. The CG and CC genotype classes were not significantly different from each other, showing full dominance. The estimated genetic variance explained by the predicted causal variant was between 7.3 and 15.8% for KLH-binding IgG NAb, KLH-binding IgT NAb, and tIgM, and was 63.5% for KLH-binding IgM NAb. KLH-binding IgA NAb, tIgA, tIgG, and tIgT were not significantly associated.

**Table 5:** Significance, and effects of the predicted causal variant (SNP on chromosome 4 position 69,965,939 bp, based on Gallus_gallus-5.0) of KLH-binding IgM natural antibody (NAb) (IgM) titer, and total IgM (tIgM) concentration (μg/ml) on all measured KLH-binding NAb titers, and total antibody concentrations (μg/ml) in an adolescent White Leghorn chicken population.

Trait	*p-Value*	GG (SE)^a^^b^	CG^a^^b^	CC (SE)^a^^b^	$\sigma_{\text{SNP}}^{\text{2}}∕\sigma_{\text{a}}^{\text{2}}$ (%)
IgM	<0.001	−0.61^y^ (0.06)	0^z^	0.04^z^ (0.06)	63.5
IgA	0.45				
IgG	0.05	−0.11 (0.08)	0	0.15 (0.09)	7.3
IgT	<0.001	−0.35^y^ (0.08)	0^z^	0.08^z^ (0.08)	15.8
tIgM	<0.001	−39.31^y,^^c^	0^z^	6.64^z,^^c^	13.3
tIgA	0.84				
tIgG	0.26				
tIgT	0.70				

*Analyzed traits were KLH-binding IgM NAb (IgM) titer, KLH-binding IgA NAb (IgA) titer, KLH-binding IgG NAb (IgG) titer, total KLH-binding NAb (IgT) titer, total IgM (tIgM) concentration, total IgA (tIgA) concentration, total IgG (tIgG) concentration, and total antibody (tIgT) concentration. tIgM, tIgA, tIgG, and tIgT were log_10_-tranformed for the analyses. Only significant SNP effects are reported. The estimated genotype effects for tIgM, tIgA, tIgG, and tIgT are reported in μg/ml. The table shows: trait, *p*-value, effect of genotype classes (and SE) in titer points for NAb, or μg/ml for total antibody concentrations, and percentage of additive genetic variance explained by the investigated SNP ($\sigma_{\text{SNP}}^{\text{2}}∕\sigma_{\text{a}}^{\text{2}}\times 100\%$). Genotype classes with significantly (*p*-value ≤ 0.05) different effects are indicated by different superscripts (y or z)*.

*^a^Genotype effects are compared to the CG genotype class, which was set to 0 (zero)*.

*^b^The number of observations per genotype class varied between 457 and 475 for GG; 803 and 831 for CG; and 313 and 321 for CC*.

*^c^The estimated effects are reported in μg/ml but were calculated from the predicted values on a log_10_-scale. Predicted values were 2.43 for GG, 2.49 for CG, and 2.50 for CC. Estimated effects based on log_10_-analysis were −0.06 (0.01) for GG and 0.01 (0.01) for CC*.

## Discussion

Relatively few GWAS have focused on detecting genomic regions associated with immunity in chicken (3, 34, 35). Though two KLH-binding NAb association studies with dedicated SNP sets were conducted across chicken lines (13, 14). To the best of our knowledge, this study is the first genome-wide association study on (KLH-binding) NAb levels in chickens. None of previously associated genes (13, 14) could be confirmed in this study, likely because of the high genetic uniformity in our data set as a result of a within line study instead of an across line study. No other study found the associated region on chromosome 4 reported in this study, which might indicate that this association is unique for this chicken line. This is also the first study that investigated heritabilities of total antibody concentrations or performed GWAS on all total antibody isotype concentrations in chickens.

57,636 SNP were used to perform the GWAS. 73.0% of the SNP were non-informative, mainly because the SNP were fixed (62.2%). This is in agreement with previously reported genetic uniformity in White Leghorn layer chicken lines (36). In total 15,580 SNP passed quality control. For five out of eight analyzed traits population stratification was found to be present, which suggests family structures within the studied population that are not captured by the pedigree information. Other factors, like heterogeneous variance, or model misspecification, likely also contributed to this, since inflation factors were not of equal size among the traits. Especially for KLH-binding IgM NAb, the population stratification was high. After correcting the test statistics for the inflation factor λ, the new inflation factor indicated that KLH-binding IgM NAb was overcorrected (λ = 0.88). Thereby being possibly too conservative on the associations with KLH-binding IgM NAb.

The heritability of tIgM was low, but twice the heritability of KLH-binding IgM NAb (4). KLH-binding IgM NAb have been found to be influenced by maternal environmental effects (4). In case maternal effects are not accounted for in genetic analyses, heritabilities will be overestimated as was seen for KLH-binding IgM NAb (4). The size of the present data set did not allow detection of maternal environmental effect for KLH-binding IgM NAb. The estimated heritability (without maternal environmental effect) for IgM NAb in this study population was 0.31 (data not shown), which is similar to the heritability of tIgM. In addition, the genetic correlation between KLH-binding IgM, and tIgM was very high. This suggests that maternal environmental effects for tIgM are present, but could not be estimated, and resulted in overestimation of tIgM heritability.

One significant genomic region was identified for KLH-binding IgM NAb, and tIgM on chromosome 4 around 70 Mbp. When correcting KLH-binding IgM NAb, and tIgM data for the estimated effects, the genomic region disappeared (data not shown). This shows that only one genomic region (i.e., one gene with one causal variant) is responsible for the observed effects.

All of the candidate genes in the IgM-associated region on chromosome 4 have been related to organism development, cell cycle, cell proliferation, metabolism, cancer development, viral infections, or a combination (mostly based on homologues in human and mouse studies), except for *SMIM14*, *TMEM156*, *FAM114A1* (no literature available) (31). This implies that all candidate genes have a potential to influence IgM antibodies. However, some genes are reported in association with antibodies, or B cells, which makes them more likely candidates: *KLF3* knockout mice were reported to have impaired B cell differentiation, including NAb-producing B1 B cells (37). TLR, including TLR1/TLR6 [homolog/paralog to TLR1A/TLR1B (38)], are well-known for their essential role in antibody production after stimulation of B cells, including B1 B cells (39–41). TBC1D1 has been reported to have a potential downstream role of silencing of pre-BCR signaling in acute lymphoblastic leukemia in human (42). However, none of these studies reported an effect on IgM without deliberate stimulation of B cells in healthy individuals, as is the case in this study.

Fine mapping of the associated region allowed to predict changes in protein functioning as a result of nucleotide changes in coding regions. A nucleotide change within *TLR1A* was predicted to have severe impact on protein folding/functioning, making this SNP the most likely candidate for the major effects on KLH-binding IgM NAb, tIgM, and KLH-binding IgT NAb. TLR1A, also known as TLR1-like a (TLR1La), TLR1.1, TLR1/6/10, and TLR16 (43), is one of the 10 known chicken TLR [see Brownlie and Allan (44) for a review on avian TLR]. TLR are among the most studied immune receptors. They are a family of transmembrane proteins that recognize conserved molecular patterns (pathogen-/microbe-associated molecular patterns; PAMP/MAMP), and are conserved in evolution (45). TLR1A dimerizes with TLR2 (either TLR2A or TLR2B), and recognizes peptidoglycans and related structures, including FSL-1, and PAM_3_CSK_4_ (33, 46), though some anomalies exist between studies (44). TLR1A contains 19 LRRs (33), of which LRR6-16 are required for ligand specificity (33). The region close to the C-terminal end of TLR1A is required for dimerization with TLR2 (in humans) (47, 48). The candidate SNP is located in LRR4 near the N-terminal end, suggesting not a direct influence on ligand recognition, or dimerization. Instead, it is likely influencing the tertiary protein structure, or mediation of coreceptors with TLR [see Van Bergenhenegouwen et al. (49) for a review], or both. Also, only coding regions were checked for amino acid substitutions, but not, for example, promotor regions influencing expression of genes. Based on the data in this study, no conclusion can be drawn on whether the most likely causal variant results in a complete loss of function, or in reduced functioning. Nevertheless, full dominance was observed for this *TLR1A* variant, suggesting that one functional copy of the gene results in a sufficient expression of functional TLR1A heterodimer and enhancement by the coreceptors for activation. Future studies should confirm the predicted *TLR1A* variant and its functionality.

The most likely causal variant has an effect on KLH-binding IgM NAb, and tIgM. The same region was found for IgM binding the self-antigen cardiolipin, and IgM binding the self-antigen ovalbumin (Bao et al., submitted). It is tempting to speculate that this *TLR1A* variant has a role on maturation/proliferation/survival of naive (NAb-producing) B cells, (KLH-binding) IgM production, or a combination of these. The most likely causal variant can affect the three stages of the (avian) B cell development: prebursal (only before hatch), bursal, and postbursal (50). Considering *TLR1A* as the most likely candidate, the effect on B cells, and IgM antibody levels is expected after exposure to PAMP, i.e., after hatch. *TLR1A* is expressed on various types of cells, including macrophages, and B cells, and in various organs, including thymus, spleen, liver, and bursa of Fabricius (44, 51). The effect of the *TLR1A* variant on IgM (NAb) can therefore be either directly on B cells or indirectly on B cells *via* for example macrophages, or both. An effect of the *TLR1A* variant is also expected on other cells of the immune system, both of the innate and adaptive part. This *TLR1A* variant is of particular interest, because it might explain part of the association of especially IgM NAb at adolescence with survival in chicken (11, 12). Yilmaz et al. (52) suggested that the TLR1 and TLR2 subfamilies are under positive selection (favoring polymorphisms) in chicken, while all other TLR are under negative/purifying selection (favoring conserved structures) (52). Huang et al. (38) suggested that only certain sites of TLR1 might be under positive selection, but that TLR1 is mostly under purifying selection (38). Nevertheless, the results imply that *TLR1A* is not essential for healthy chickens, but that it does influence IgM levels.

The identified region on chromosome 4 has previously been associated with many other characteristics in chickens (layer, broiler, or both) [based on Animal QTLdb (53)]. Only one of these are directly related to disease susceptibility: for susceptibility to Marek’s disease virus (54). Though the association with Marek’s susceptibility was stronger around TLR3 (also located on chromosome 4, around 62 Mbp), which is involved in the recognition of viruses, including Marek’s disease virus infections (55). Other characteristics associated with these region were for example age at first egg (56), eggshell breaking strength (57), eggshell color (58, 59), egg weight (57, 60, 61), feed intake (60), growth/body weight at several ages (56, 62–68), liver weight (69), and spleen weight (70). To investigate these associations, production traits of 222 females of the study population were previously collected [described in van der Klein et al. (15)], and could therefore be tested for their association with the most likely causal variant. Of the investigated traits, only the number of laid eggs between 17 and 24 weeks of age (i.e., adolescence) tended (*p* = 0.09) to a significant association with the *TLR1A* variant, where the genotype associated with lower IgM levels was associated with higher egg production (data not shown). Such a beneficial effect of the *TLR1A* variant on one of the most important production traits in layer chickens, in combination with a reduced natural selection pressure (due to high sanitary barriers on the breeding nucleus), might explain why the variant is present in the study population, even though it might impair (humoral) immunity.

The heritability of tIgA was twice as large as the heritability of KLH-binding IgA NAb. Interestingly, similar concentrations for tIgA, and tIgM were found, which is the first time to be reported. Genetic, and phenotypic correlations between KLH-binding IgA NAb and tIgA were positive, and low to moderate. This suggests that variation in KLH-binding IgA NAb, and tIgA are mainly regulated by different mechanisms. The role of blood IgA antibodies is not well understood so far. It has been suggested to be a response isotype with a proinflammatory function, similar to IgG antibodies (41, 71, 72). However, in chicken most of the intestinal IgA is secreted *via* the bile (73, 74), which means that intestinal IgA first has to be transported through the blood. IgA in blood might therefore represent the intestinal health status in chickens.

Two suggestively associated regions were identified for KLH-binding IgA NAb. These regions were not confirmed by tIgA concentration, even though a positive genetic correlation was estimated. This is the first time an association study on IgA (either NAb or total antibody concentration) in healthy individuals has been done. Observed results on KLH-binding IgA NAb were suggestive, and therefore these results first need to be confirmed in independent studies before further investigation of these associations.

Heritabilities of tIgG, and tIgT antibody concentrations were low, but comparable to heritabilities of KLH-binding IgG NAb titers, and KLH-binding IgT NAb titers (4). This indicates a strong environmental influence on IgG antibodies, and consequently on IgT, since IgT mainly consists of IgG antibodies: all genetic, and phenotypic correlations were higher than 0.8 [this study and Berghof et al. (4)]. However, KLH-binding IgT NAb, but not tIgT, was suggestively associated with the same region of KLH-binding IgM NAb, and tIgM. This confirms the high genetic correlation between KLH-binding IgT NAb, and KLH-binding IgM NAb (4), and the moderate genetic correlation between tIgT, and tIgM (this study).

Remarkably the genetic correlations between KLH-binding IgG NAb, and tIgG, and KLH-binding IgT NAb, and tIgT were negative. This suggest that KLH-binding IgG NAb, and tIgG, and KLH-binding IgT NAb, and tIgT are genetically regulated in a similar way, but with opposing effects. However, phenotypically there were very weak correlations between KLH-binding IgG NAb, and tIgG, and KLH-binding IgT NAb, and tIgT. In addition KLH-binding IgG NAb, and KLH-binding IgT NAb titers were significantly associated with the *TLR1A* variant, but tIgG, and tIgT concentrations were not. Meaning that a genetic predisposition for low (or high) levels of KLH-binding IgG NAb, and KLH-binding IgT NAb, or different genotypes for the *TLR1A* variant do not negatively (or positively) affect the tIgG antibody concentrations, and tIgT antibody concentrations. This implies that the humoral adaptive immune response, and memory formation are not negatively influenced by low NAb levels, or the *TLR1A* variant.

Natural antibodies are defined as antigen binding antibodies present in individuals without a previous exposure to this antigen (5). Layer chickens receive an extensive number of obligatory vaccinations, that may affect antibody levels in a (non-specific) fashion. In addition, although thought to be unlikely, KLH-binding antibodies could have been generated due to cross-reactivity with a component(s) of the vaccination(s). Also, due to KLH’s molecular weight (75), cross-reactivity of KLH-binding NAb with other antigens cannot be excluded. For example, cross reactivity of antibodies binding KLH with antigens of the tropical parasitic worm *Schistosoma mansoni* has been suggested (76). This might suggest that NAb can be made in the presence of exogenous antigens, which is currently under investigation [e.g., Baumgarth (77)]. Nevertheless, the whole study population received the same vaccinations, and a possible effect of moment of blood sampling (between 15 and 22 weeks of age) was corrected by the regression coefficient of “age” in the statistical model. Therefore, the vaccinations likely do not underlie the observed results.

In this study, we show the potential of using a large database with defined immunological traits to discover underlying genomic regions, and new fundamental components in the development, and functioning of the immune system. Recently in humans, similar approaches have been used to detect genetic variation for several immune traits, not only measuring baseline immunity but also functional immunological assays (78, 79). GWAS for defined immunological traits offer exiting, and promising opportunities for unraveling genetic variation, and fundamental mechanisms in the immune system in human, and animal species.

In summary, in this GWAS for KLH-binding NAb, and total concentrations of antibodies, we identified one significantly associated region on chromosome 4 with KLH-binding IgT NAb, total IgM concentration, and especially with KLH-binding IgM NAb. Further investigation predicted the causal variant on chromosome 4 to be located in *TLR1A*. As far as we know, this is the first time a TLR has been connected to IgM antibody levels in healthy individuals. This suggests a fundamental role of TLR in KLH-binding IgM NAb production, total IgM production, and possibly in (NAb-producing) B cell development, and proliferation. Future research should confirm the role of *TLR1A*, its role in KLH-binding IgM production, and total IgM production, and its role in general disease resistance in chickens.

## Ethics Statement

Collection of samples and data was done according to Hendrix Genetics (HG) protocols, under the supervision of HG employees. Samples and data were collected as part of routine data collection in a commercial breeding program for layer chickens in The Netherlands. Samples and data were collected on a breeding nucleus of HG for breeding purposes only, and are a non-experimental, agricultural practice, regulated by the Act Animals, and the Royal Decree on Procedures. The Dutch Experiments on Animals Act does not apply to non-experimental, agricultural practices. An ethical review by the Statement Animal Experiment Committee was therefore not required. No extra discomfort was caused for sample collection for the purpose of this study.

## Author Contributions

TB performed the research, analyzed, and interpreted the data, and wrote the manuscript. MV and JA analyzed and interpreted the data, and revised the manuscript. HP, JP, and HB designed the research, interpreted the data, and critically revised the manuscript. AV performed the research and analyzed and interpreted the data.

## Conflict of Interest Statement

This study was in kind financed by Hendrix Genetics. In addition, AV is employed by Hendrix Genetics Research. AV was involved in genotyping and sequencing of the chickens and imputation of genomic data to the described genomic data sets. These genomic data sets are also of interest for Hendrix Genetics’ commercial targets, but this interest did not influence the results in this manuscript in any matter. Except for the delivered data, and the results reported in this project, or for other projects, no other shared interests (e.g., employment, consultancy, patents, products) exist between Hendrix Genetics, and Wageningen University & Research. All other authors declare that the research was conducted in the absence of any commercial or financial relationships that could be construed as a potential conflict of interest.

## Footnotes

^1^http://www.fao.org/ag/againfo/themes/en/poultry/animal_health.html.

^2^See also http://www.antibodyregistry.org.
