# Supplementary material for: Genomic Region Containing Toll-Like Receptor Genes Has a Major Impact on Total IgM Antibodies Including KLH-Binding IgM Natural Antibodies in Chickens
Source: Front Immunol. 2018 Jan 9;8:1879. doi: 10.3389/fimmu.2017.01879 (PMC5767321; doi:10.3389/fimmu.2017.01879)
Supplement: Supplementary file 1 [file Table_1.PDF]

Supplementary Material

Genomic Region Containing Toll-like Receptor Genes Has a Major Effect on IgM (Natural) Antibodies in Chickens

T.V.L. Berghof*, M.H.P.W. Visker, J.A.J. Arts, H.K. Parmentier, J.J. van der Poel, A.L.J. Vereijken, H. Bovenhuis

* **Correspondence:** Corresponding Author: tom.berghof@wur.nl

Supplementary Figures and Tables

Supplementary Table 1.

Distribution of SNP used in this study over the chicken genome. The table shows: name of the chromosome (chromosomes 1-28 and 30-33, linkage group LGE64, sex chromosomes W and Z, and unplaced), total length of the chromosome (in basepair), the number of SNP on the chromosome, the number of monomorphic SNP (fixed) removed after quality control (step 1), the number of SNP removed after quality control (step 2-4), the number of SNP used in the genome-wide association studies. Table is based on Gallus_gallus-5.0.

		# SNP			
**Name**	**Total length (bp)**	**All**	**Fixed**[a]	**Cleaned**	**Used**
1	210,673,733	8,651	5,470	757	2,424
2	158,710,615	6,574	4,418	433	1,723
3	116,920,747	4,953	3,261	523	1,169
4	96,543,418	4,036	2,336	358	1,342
5	63,034,728	2,640	1,660	216	764
6	37,500,173	2,089	1,115	179	795
7	38,707,053	2,177	1,332	187	658
8	32,086,491	1,700	898	149	653
9	25,237,187	1,426	770	106	550
10	21,385,625	1,618	941	187	490
11	21,449,822	1,577	1,028	146	403
12	21,002,089	1,640	1,126	232	282
13	19,897,466	1,437	1,028	101	308
14	16,681,244	1,234	736	105	393
15	13,353,506	1,258	940	131	187
16	1,058,524	35	20	5	10
17	11,686,891	1,055	646	163	246
18	11,426,502	1,087	734	129	224
19	10,716,916	1,011	467	137	407
20	15,720,949	1,869	1,246	118	505

Supplementary Table 1. continued

Name	Total length (bp)	# SNP			
		**All**	**Fixed**[a]	**Cleaned**	**Used**
21	7,159,517	936	522	103	311
22	5,435,368	473	319	45	109
23	6,289,313	761	401	119	241
24	6,631,702	886	510	89	287
25	3,644,590	240	169	10	61
26	5,546,572	827	502	72	253
27	6,304,838	613	423	34	156
28	5,243,714	796	522	42	232
30	224,321	0	-	-	-
31	168,864	0	-	-	-
32	253,552	0	-	-	-
33	3,756,441	0	-	-	-
LGE64	1,217,975	39	28	1	10
W	7,082,455	11	11	0	0
Z	88,942,393	3,058	2,966	88	333
unplaced	138,546,488	929	508	40	52
Total	1,230,241,782	57,636	37,053	5,005	15,578

[a] Monomorphic SNP
