# Supplementary material for: Genomic Region Containing Toll-Like Receptor Genes Has a Major Impact on Total IgM Antibodies Including KLH-Binding IgM Natural Antibodies in Chickens
Source: Front Immunol. 2018 Jan 9;8:1879. doi: 10.3389/fimmu.2017.01879 (PMC5767321; doi:10.3389/fimmu.2017.01879)
Supplement: Supplementary file 2 [file Table_2.PDF]

Supplementary Material

Genomic Region Containing Toll-like Receptor Genes Has a Major Effect on IgM (Natural) Antibodies in Chickens

T.V.L. Berghof*, M.H.P.W. Visker, J.A.J. Arts, H.K. Parmentier, J.J. van der Poel, A.L.J. Vereijken, H. Bovenhuis

*** Correspondence:** Corresponding Author: tom.berghof@wur.nl

Supplementary Figures and Tables

Supplementary Table 2.

Estimated genetic correlations (below the diagonal), and phenotypic correlations (above the diagonal) of total IgM (tIgM) concentration, total IgA (tIgA) concentration, total IgG (tIgG) concentration, and total antibody (tIgT) concentration in a WA leghorn chicken population at adolescence. tIgT, tIgM, tIgA, and tIgG were $\log_{10}$-tranformed for the analyses. SE are shown in parentheses.

	**tIgM**	**tIgA**	**tIgG**	**tIgT**
**tIgM**	-	0.18 (0.03)	0.29 (0.02)	0.39 (0.02)
**tIgA**	0.17 (0.18)	-	0.13 (0.03)	0.19 (0.03)
**tIgG**	0.12 (0.26)	0.62 (0.23)	-	0.99 (0.0003)
**tIgT**[a]	0.24 (0.22)	0.72 (0.19)	0.99 (0.01)	-

[a] tIgT = tIgM + tIgA + tIgG
