# Supplementary material for: Genomic Region Containing Toll-Like Receptor Genes Has a Major Impact on Total IgM Antibodies Including KLH-Binding IgM Natural Antibodies in Chickens
Source: Front Immunol. 2018 Jan 9;8:1879. doi: 10.3389/fimmu.2017.01879 (PMC5767321; doi:10.3389/fimmu.2017.01879)
Supplement: Supplementary file 3 [file Table_3.PDF]

Supplementary Material

Genomic Region Containing Toll-like Receptor Genes Has a Major Effect on IgM (Natural) Antibodies in Chickens

T.V.L. Berghof*, M.H.P.W. Visker, J.A.J. Arts, H.K. Parmentier, J.J. van der Poel, A.L.J. Vereijken, H. Bovenhuis

*** Correspondence:** Corresponding Author: tom.berghof@wur.nl

Supplementary Figures and Tables

Supplementary Table 3.

All suggestively or significantly associated SNP within genomic regions identified in genome-wide association studies for KLH-binding natural antibody (NAb) titers and total antibody concentrations (µg/ml) in a WA leghorn chicken population around 16 weeks of age. Genomic regions were associated for total KLH-binding NAb (IgT) titer, KLH-binding IgM NAb (IgM) titer, total IgM (tIgM) concentration, and KLH-binding IgA NAb (IgA) titer. The table shows: chromosome of associated genomic region, position of significantly or suggestively associated SNP (in basepair), name of the SNP, the $-\log_{10}$(p-value) of the SNP with the associated trait, and the SNP set used to genotype. Only significant (FDR = 0.05) or suggestive (FDR = 0.20; *italic*) $-\log_{10}$(p-value)'s are reported. Positions are based on Gallus_gallus-5.0.

**Chromosome**	**Position (bp)**	**Name**	**IgT**	**IgM**	**tIgM**	**IgA**	**SNP set[a]**
4	69,587,709	rs313004783	*5.21*	13.21	6.55		11k
	69,702,481	rs15614874	*4.79*	13.84	6.36		imputed
	69,703,708	rs14488032		11.62	5.38		imputed
	69,814,286	rs313437715	*4.61*	12.79	6.90		11k
	69,951,230	rs16742890		10.16			11k
	70,086,585	rs14696113		8.74			imputed
	70,109,615	rs14694747		5.23	*4.57*		imputed
	70,220,795	rs16756059		8.39			imputed
	70,250,789	rs80589947		8.33			imputed
	70,353,267	rs14488185		6.59			11k
	70,670,047	rs15615299		6.56			11k
	70,689,865	rs16433183		6.04			imputed
	70,769,109	rs14488433		6.11			11k
	71,057,565	rs15615517		6.24			imputed
	71,097,736	rs16433426		5.47			3k+11k
	71,140,058	rs16433455		6.47			imputed
	71,156,953	rs16433473		5.68			imputed
	71,519,305	rs15615695		5.71			imputed
	71,531,594	rs14488824		5.76			imputed

Supplementary Table 3. continued

**Chromosome**	**Position (bp)**	**Name**	**IgT**	**IgM**	**tIgM**	**IgA**	**SNP set[a]**
	71,557,148	rs13774302		5.49			11k
	71,585,796	rs14488914		5.78			imputed
	71,591,683	rs13774314		5.47			11k
	71,626,662	rs14488949		5.78			imputed
	71,684,776	rs14488121		5.63			11k
	71,804,632	rs16433788		5.68			imputed
	71,869,873	rs14489012		5.68			imputed
	71,910,272	rs16433853		5.82			imputed
	71,934,160	rs14489032		5.54			imputed
	71,989,867	rs14489048		5.54			imputed
	72,181,481	rs16433969		6.34			imputed
	72,517,503	rs15616020		6.34			imputed
	72,529,232	rs317821928		6.34			imputed
	72,548,431	rs312660474		6.34			imputed
	73,343,240	rs15616529		5.44			imputed
	73,362,701	rs316906071		5.19			11k
9	12,230,193	rs14672112				*4.34*	11k
	12,261,658	rs15969591				*4.58*	imputed
18	9,984,590	rs16347444				*4.55*	imputed
	10,131,465	rs10731438				*4.55*	11k
	10,154,530	rs14417110				*4.55*	imputed

[a] SNP genotyped with a 2,740 (3k) SNP set, a 11,173 (11k) SNP set, or imputed.
